# Comparative gallium-68 labeling of TRAP-, NOTA-, and DOTA-peptides: practical consequences for the future of gallium-68-PET

**DOI:** 10.1186/2191-219X-2-28

**Published:** 2012-06-09

**Authors:** Johannes Notni, Karolin Pohle, Hans-Jürgen Wester

**Affiliations:** 1 Pharmaceutical Radiochemistry, Technische Universität München, Walther-Meissner-Str. 3, Garching, 85748, Germany; 2 Department of Nuclear Medicine, Klinikum rechts der Isar, Technische Universität München, Ismaninger Str. 22, Munich, 81675, Germany

**Keywords:** macrocyclic ligands, gallium-68, positron-emission tomography, peptides, bioconjugates, radiolabeling

## Abstract

**Background:**

Currently, ^68^Ga-labeled 1,4,7,10-tetraazacyclododecane-tetraacetic acid (DOTA)-peptides are the most widely used class of ^68^Ga radiotracers for PET, although DOTA is not optimal for ^68^Ga complexation. More recently, 1,4,7-triazacyclononane-triacetic acid (NOTA) and particularly triazacyclononane-phosphinate (TRAP) chelators have been shown to possess superior ^68^Ga binding ability. Here, we report on the efficiency, reproducibility, and achievable specific activity for fully automated ^68^Ga labeling of DOTA-, NOTA-, and TRAP-peptide conjugates.

**Findings:**

Compared to NOTA- and DOTA-peptides, achievable specific activity (*A*_S_) for TRAP-peptide is approximately 10 and 20 times higher, respectively. *A*_S_ values in the range of 5,000 GBq/μmol were routinely obtained using 1 GBq of ^68^Ga, equivalent to 0.11 μg of cold mass for a 185-MBq patient dose of a 3-kDa conjugate. The TRAP-peptide could be ^68^Ga-labeled with excellent reproducibility and > 95% radiochemical yield for precursor amounts as low as 1 nmol.

**Conclusions:**

High ^68^Ga labeling efficiency of TRAP-peptides could facilitate realization of kit labeling procedures. The good reproducibility of the automated synthesis is of relevance for GMP production, and the possibility to provide very high specific activities offers a high degree of safety in first clinical trials, due to reduction of cold mass content in tracer formulations.

## Findings

### Background

With the commercial availability of ^68^Ge/^68^Ga generators, cyclotron-independent on-site production of tracers for positron-emission tomography (PET) has become widely feasible [1,2]. Thus, in the near future, a ubiquitous implementation of PET and PET/CT even in regions with less well-developed infrastructure can be expected, similar to the global story of success of ^99m^Tc-based scintigraphy which started half a century ago with the introduction of ^99^Mo/^99m^Tc generators [1,3]. In the long run, a partial substitution of single photon emission computed tomography (SPECT) by PET (and SPECT/CT by PET/CT, respectively) appears to be a realistic scenario in view of the advantages of PET, such as superior spatial resolution and sensitivity. Besides, in contrast to reactor-produced ^99^Mo, ^68^Ge is cyclotron-produced. This can be considered advantageous with regard to the recent insufficiency of global reactor capacity for reliable ^99^Mo supply [4], and independence of ^68^Ga-PET from nuclear reactors might positively influence the bias of its public perception.

Generally, ^68^Ga labeling is done by complexation of the ^68^Ga^3+^ ion. For this purpose, dedicated chelators usually have to be introduced into precursor molecules by bioconjugation, wherein they readily determine the labeling chemistry. To facilitate global implementation of ^68^Ga-PET, production of ^68^Ga radiopharmaceuticals must be simple, robust, and reliable; this demands highly efficient labeling chemistry and, therefore, highly efficient chelators. Recently, we have shown that the bifunctional triazacyclononane-phosphinate (TRAP) ligand [5-8] possesses markedly improved ^68^Ga labeling properties [6]. This applies also to TRAP-based peptide conjugates, the practical consequences of which we further elucidate in this study.

### Methods

TRAP(RGD)_3_ was prepared as described before [6]. NODAGA-cyclo(RGDyK) (‘NODAGA-RGD’) was purchased from ABX GmbH (Radeberg, Germany). DOTATOC was obtained from Bachem (Bubendorf, Switzerland). Fully automated ^68^Ga labeling was performed using unpurified eluate fractions of a ^68^Ge/^68^Ga generator with SnO_2_ matrix (iThemba LABS, Somerset West, South Africa), as described previously [6,9] (5 min reaction at 95°C, pH adjusted with HEPES, pH 3.2 for DOTATOC and NODAGA-RGD, pH 2 for TRAP(RGD)_3_, purification using C8 SPE cartridge). Radiochemical yield was calculated from decay-corrected product activity in relation to the sum of significant decay-corrected residual activities contained elsewhere, that is, in reaction vial, SPE cartridge, and non-product cartridge purging liquids.

### Calculation of specific activities

Product activities (*A*_P_) were measured after the end of preparation (approximately 15 min after the start of syntheses) and decay corrected to a typical injection time, 30 min after the start of synthesis (*A*_P,30_). In order to be able to calculate corresponding specific activity values that are representative for the respective precursor amounts and independent from small deviations in the starting activity *A*_0_ (in our experiments, ranging from 800 to 1,050 MBq, depending on the regeneration state of the ^68^Ga generator), product activities were normalized to a representative starting activity *A*_N_ = 1 GBq, according to $A_{\text{P},30,\text{N}}=A_{\text{P},30}\left(\frac{A_{\text{N}}}{A_{0}}\right)$. Specific activity (*A*_S_) values were calculated by the division of *A*_P,30,N_ by the precursor amount used. It is assumed that all precursor peptide is actually retained on and subsequently eluted from the cartridge, and thus transferred into the formulation. This means that both retention and elution efficiency are considered 100%, both of which can be somewhat lower in practice. As a result, all given *A*_S_ represent the lower bounds and will never overestimate actual values.

### Results and discussion

**Figure 1:**
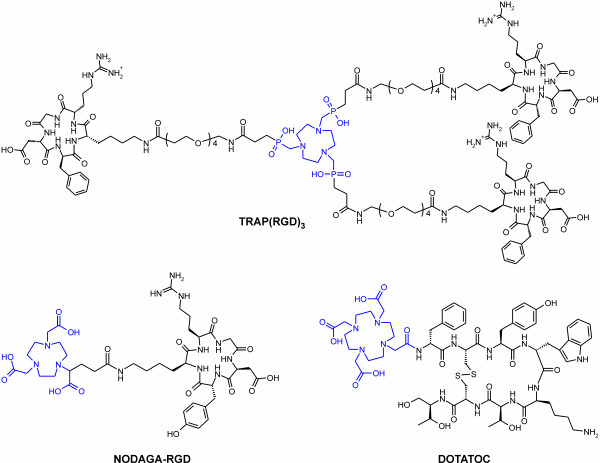
**Structures of peptide conjugates used in this study.** The complexation sites of TRAP, NOTA, and DOTA-monoamide, featured in TRAP(RGD)_3_, NODAGA-RGD, and DOTATOC, respectively, are highlighted in blue color.

Although previous comparative studies focusing on the basic chelator structures TRAP, 1,4,7-triazacyclononane-triacetic acid (NOTA), and 1,4,7,10-tetraazacyclododecane-tetraacetic acid (DOTA) already proved superior Ga^3+^ complexation/^68^Ga labeling properties of TRAP [6,7], these data are not sufficient to quantify the behavior of respective peptide conjugates, for two reasons: Firstly, functionalization of chelators with peptides, resulting in conjugates with a multiple of the molecular weight of the neat chelators, is definitely prone to change overall complexation properties with potentially unpredictable outcome. Secondly, the chelating moiety in compounds commonly dubbed ‘DOTA-peptides’ is actually not DOTA, but DOTA-monoamide (see Figure 1), which exhibits different Ga^3+^ complexation behavior [10]. In contrast, for TRAP and the bifunctional NOTA-derivative NODAGA, the structure of the chelating site is not affected by conjugation. To assess the impact of these effects, representative peptide conjugates (TRAP(RGD)_3_[6] and the commercially available ‘NOTA’- and ‘DOTA’-peptides NODAGA-RGD and DOTATOC, respectively, see Figure 1) were labeled under similar conditions using our standard automated procedure [6,9].

**Figure 2:**
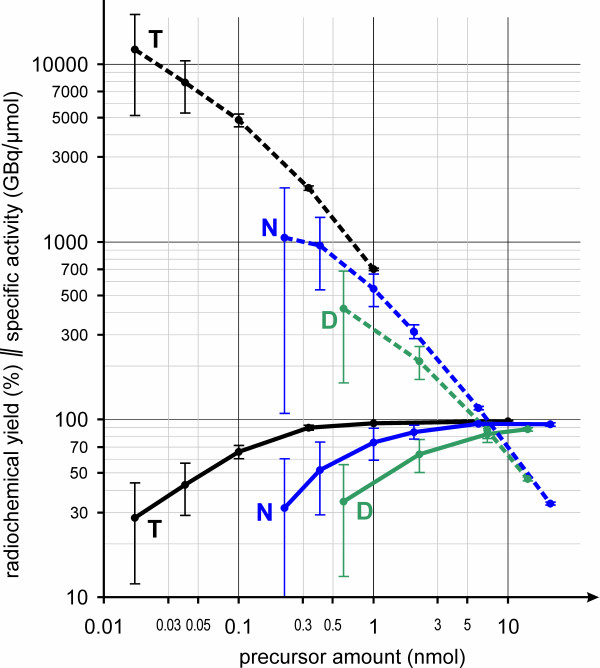
**Radiochemical yields and corresponding calculated minimal *A*_S_ of radiopharmaceutical formulations.** Radiochemical yields (solid lines, %, mean ± SD, *n* ≥ 4) and corresponding calculated minimal *A*_S_ (dashed lines, GBq/μmol, mean ± SD) of the formulations at typical time of injection (30 min after the start of synthesis) as functions of precursor amount for automated ^68^Ga labeling of TRAP(RGD)_3_ (T), NODAGA-RGD (N), and DOTATOC (D). *A*_S_ for TRAP(RGD)_3_ concentrations > 1 nmol are not shown for clarity of presentation.

Figure 2 shows that TRAP(RGD)_3_ allows to use much lower precursor concentrations for labeling than required for NODAGA-RGD and particularly DOTATOC, which is why ^68^Ga-TRAP(RGD)_3_ can be prepared with much higher *A*_S_ (see also Table 1). Using 0.1 nmol of TRAP(RGD)_3_, almost 5,000 GBq/μmol was reached with a satisfying decay-corrected yield of 66 ± 6%. The use of even lower amounts of TRAP(RGD)_3_ (17 pmol) frequently resulted in preparations with extremely high *A*_S_ of >10,000 GBq/μmol, although not reliably reproducible. The highest *A*_S_ value observed during these experiments was 14,900 GBq/μmol (actual value, not normalized to starting activity), which is approximately 1/7 of the theoretically possible maximum value, that of carrier-free ^68^Ga. Although such high specific activities are not usually needed for clinical applications, we nevertheless, deem this feature of high practical value for the following reasons:

1. A hypothetical patient dose of 185 MBq (5 mCi) of a 5,000-GBq/μmol preparation contains only 37 pmol of peptide; for a compound like TRAP(RGD)_3_ with a molecular weight of ≈ 3 kDa, this calculates to a total of 0.11 μg of cold mass, or less than 2 ng/kg body weight for an average patient. Such tiny amounts are extremely unlikely to cause any pharmacological effects. Therefore, TRAP could facilitate the use of such biomolecules for imaging that possess very high pharmacological potential, and ^68^Ga-labeled TRAP conjugates could generally offer high safety when tested in first clinical trials.

2. As a 15-MBq dose of said preparation is equivalent to 3 pmol or 9 ng of our exemplary 3-kDa peptide, it can always directly be used for evaluation studies in rodents without having to separate off unlabeled precursor or, unfavorably, reduce the administered dose. High receptor occupancy or even saturation effects, which otherwise are frequently encountered in small animal imaging due to the necessity of applying much higher activity doses per kilogram body weight than in humans, can be practically ruled out.

3. Several studies have outlined that the amount of co-injected cold mass can have a significant influence on biodistribution and imaging results [11-14]. In clinical routine, it is therefore highly recommended to utilize radiopharmaceutical formulations with constant, optimized specific activity (i.e., well-defined cold mass content). Such productions could be done most conveniently and reliably by adding the desired amount of active compound to a pre-conditioned vial containing a fixed amount of cold standard. This approach, however, requires radiolabeled tracers with very high specific activity in order not to change the overall contained amount of cold mass significantly. ^68^Ga-labeled TRAP conjugates appear ideally suited for this purpose.

**Table 1:** Radiochemical yields of automated ^68^Ga labeling, and corresponding calculated minimal *A*_S_ of radiopharmaceutical formulations

**Precursor amount (nmol)**	**Product yield (%)**	**Specific activity (GBq/μmol)**
^68^Ga-TRAP(RGD)_3_		
0.017	27.9 ± 16.0	12059 ± 6922
0.04	42.9 ± 14.0	7889 ± 2567
0.1	65.8 ± 5.6	4848 ± 414
0.33	90.0 ± 2.7	2006 ± 61
1	95.2 ± 1.7	701 ± 12
10	97.8 ± 0.5	73 ± 0.4
^68^Ga-DOTATOC		
0.6	34.4 ± 21.3	422 ± 261
2.2	63.7 ± 13.5	213 ± 45
7	82.8 ± 8.7	87 ± 9
14	88.0 ± 2.1	46 ± 1.1
^68^Ga-NODAGA-RGD		
0.22	31.7 ± 28.4	1059 ± 951
0.4	51.9 ± 22.8	954 ± 419
1	74.1 ± 15.2	545 ± 112
2	85.0 ± 7.4	313 ± 28
6	94.7 ± 1.1	116 ± 1.3
20.5	93.7 ± 1.9	34 ± 0.7

At typical time of injection (30 min after start of synthesis), values given as mean ± SD.

Furthermore, regarding Figure 2, one notices that variation of radiochemical yields, reflected by the size of error bars, is the larger the lower precursor amounts are. This is because the generator eluate usually contains traces of ionic contaminants, such as Zn^2+^, Sn^4+^, Al^3+^, and Fe^3+^, the concentration of which in the individual eluates is varying. These can compete with ^68^Ga^3+^ at the chelating site of the precursor, which is naturally the more impacting on labeling yield the lower the stoichiometric excess of precursor over ^68^Ga^3+^ ion is. The error bars in Figure 2 show that except for precursor amounts exceeding 20 nmol, use of a TRAP peptide will result in a more reliable radiosynthesis, being less prone to be perturbed by variation of other parameters (reaction pH, eluate volume, trace metal contaminations, etc.). Differences in radiochemical yield and reproducibility are very pronounced for peptide amounts in the range of 1 to 10 nmol (e.g., equivalent to 1.4 to 14 μg of DOTATOC) which are frequently used in routine ^68^Ga labeling procedures; near-quantitative yields and excellent reproducibility can be expected here for a TRAP peptide. This is of high relevance for routine GMP tracer production, where reproducibility and robustness of procedures is crucial. In addition, we assume that due to higher labeling efficiency, realization of kit labeling procedures will be much simpler using TRAP conjugates, which we deem of importance for the aforementioned possibility of global implementation of ^68^Ga-PET. Finally, the recent introduction of NOPO, a TRAP variety designed specifically for monoconjugation, expands the portfolio of P-functionalized triazacyclononane-triphosphinate chelators, thus offering even more synthetic possibilities for development of ^68^Ga tracers [15].

## Abbreviations

DOTA: 1,4,7,10-tetraazacyclododecane-tetraacetic acid; NOTA: 1,4,7-triazacyclononane-triacetic acid; PET: Positron-emission tomography; SPECT: Single photon emission computed tomography; TRAP: Triazacyclononane-phosphinate.

## Competing interests

The authors declare that they have no competing interests.

## Authors’ contributions

JN developed the study concept, performed the radiolabeling of TRAP(RGD)_3_ and DOTATOC, and wrote the manuscript. KP performed the radiolabeling of NODAGA-RGD and critically reviewed the manuscript. HJW gave advice in the interpretation of the data and critically reviewed the manuscript. All authors approved the final manuscript.
